# A phase I study of convection-enhanced delivery (CED) of liposomal-irinotecan using real-time magnetic resonance imaging in patients with recurrent high-grade glioma

**DOI:** 10.1007/s11060-024-04904-y

**Published:** 2025-01-06

**Authors:** Kazim H. Narsinh, Karishma Kumar, Krystof Bankiewicz, Alastair J. Martin, Mitchell Berger, Jennifer Clarke, Jennie Taylor, Nancy Ann Oberheim Bush, Annette M. Molinaro, Manish Aghi, Nicholas Butowski

**Affiliations:** 1 Department of Neurological Surgery, University of California San Francisco, San Francisco, CA USA; 2 Department of Radiology & Biomedical Imaging, San Francisco, CA USA; 3 Department of Neurology, University of California San Francisco, San Francisco, USA; 4 Department of Neurological Surgery, Ohio State University, Columbus, OH USA

**Keywords:** Convection enhanced delivery, CED, Nano-liposomal irinotecan, MRI, Liposomal irinotecan, High grade gliomas, Glioblastoma, Gliosarcoma, Anaplastic astrocytoma, Oligodendroglioma, Recurrent, Multiply recurrent

## Abstract

**Background:**

Irinotecan demonstrates anti-tumor efficacy in preclinical glioma models but clinical results are modest due to drug delivery limitations. Convection enhanced delivery (CED) improves drug delivery by increasing intratumoral drug concentration. Real-time magnetic resonance imaging of infusate delivery during CED may optimize tumor coverage. This phase 1 trial examines the safety and tolerability of liposomal irinotecan and gadolinium delivered via CED using real-time MRI guidance in recurrent high-grade glioma patients.

**Methods:**

Initially, a 3 + 3 dose-escalating, single dose trial was planned with 4 cohorts based on a fixed drug dose and volume. After 9 patients, a protocol amendment allowed for variable volume and dose of the study agent based on tumor size. The amended design specified ‘personalized’ drug volume but fixed concentration of 20 mg/mL of liposomal irinotecan in the first cohort escalating to 40 mg/mL in the second cohort.

**Results:**

Eighteen patients with recurrent WHO grade 3 or 4 gliomas (diameter 1–4 cm) were treated. Based on the tumor volume, the total dose of liposomal irinotecan was 20–680 mg in a total volume of 2–17 ml. Technical challenges were overcome by real-time MRI guidance and protocol amendment. The only dose-limiting toxicity (DLT) was a grade 3 stroke. Safety and survival information is presented.

**Conclusions:**

CED of liposomal irinotecan using real-time MRI in patients with recurrent high-grade glioma is feasible. Image-guidance allowed for improved placement of CED cannulas and optimal tumor coverage. Our results warrant further study with repeat CED dosing.

**Supplementary Information:**

The online version contains supplementary material available at 10.1007/s11060-024-04904-y.

## Introduction

High-grade gliomas (HGGs) comprise the most common primary brain tumors in adults, with 12,000–20,000 new cases a year [1]. The prognosis of HGG is poor, as median overall survival is only 10–12 months for patients with newly-diagnosed glioblastoma (GB) and 24–36 months for patients with anaplastic astrocytoma [2, 3]. These poor outcomes reflect the limited efficacy of chemotherapeutics, marred by (1) poor delivery across the blood-brain barrier (BBB), (2) emergence of resistance due to genetic and epigenetic clonal heterogeneity, (3) the tumor’s infiltrative nature, (4) abnormal tumor-associated angiogenesis, (5) local and systemic immunosuppression, and (6) the changing brain microenvironment. A treatment delivery strategy that can overcome these factors may improve outcomes [4]. Since HGG recurrence typically occurs within 2 cm of the original resection margin [5], improved local drug delivery can potentially impact survival.

Irinotecan is a topoisomerase I inhibitor that has demonstrated antitumor activity in preclinical glioma models and has a distinct mechanism of action [6]. However, irinotecan has complex pharmacology, requiring conversion to SN-38 for optimal activity, but must avoid inactivation via hydrolysis of the requisite lactone configuration to an inactive carboxylate. Also, hepatic conversion of SN-38 leads to biliary excretion of the potent metabolite and results in gastrointestinal toxicity. Liposomal encapsulation of irinotecan improves these pharmacokinetics and reduces systemic toxicity [7, 7]. Liposomal irinotecan is currently FDA-cleared for treatment of metastatic pancreatic adenocarcinoma after disease progression following gemcitabine-based therapy.

Convection-enhanced delivery (CED) is a method of locoregional infusion that delivers a high concentration of therapeutic compounds directly to the brain through a thin cannula attached to a microinfusion pump. Delivery of liposomal irinotecan via convection-enhanced delivery (CED) achieves high intratumoral drug concentrations and bypasses the BBB while limiting systemic toxicity [8–46]. Real-time MRI guidance during CED allows for adequate coverage of the tumor with sufficient drug and provides an opportunity to correct for suboptimal drug delivery [39, 47–53]. This phase 1 study aims to determine the safety, tolerability, and efficacy of delivery of liposomal irinotecan with gadoteridol via CED using real-time MRI guidance.

## Methods

### Patient population

Eligible patients were 18 + years of age with previously confirmed high-grade glioma {glioblastoma multiforme (GBM), gliosarcoma (GS), anaplastic astrocytoma (AA), or anaplastic oligodendroglioma (AO)}, who showed clinical and radiological evidence of recurrence. There was no limit to the number of recurrences prior to participation, but they could only have had one course of radiation, and needed radiologic evidence of progressive disease in a single, supratentorial tumor, with contrast-enhancing component having a diameter no larger than 4 cm or volume of 34 cm [3]. Progression was determined by consensus agreement after presentation at a multidisciplinary tumor board. Additional eligibility criteria were a KPS ≥ 70 and a life expectancy of > 8 weeks. MRI must have been performed within 21 days prior to treatment and patients who were receiving steroids must have been on a stable or decreasing dose for at least 5 days prior to imaging.

### Study design

The original design was a 4 cohort, 3 + 3 single dose-escalating trial of liposomal irinotecan (Onivyde ^®^) plus gadoteridol (ProHance ^®^) based on a fixed irinotecan dose and volume (Supplemental Table 1). Due to the small volume of drug (1-2mL) not affording adequate tumor coverage (tumor coverage on the 9 patients treated on the original protocol ranged from ~ 10–60%), the protocol was amended to allow for a single CED administration of liposomal irinotecan plus gadoteridol at prespecified concentrations with total volume tailored to each patient’s tumor size (Supplemental Table 2). What ensued was a phase 1 trial with two cohorts of variable drug volume but fixed concentration of 20 mg/mL, then 40 mg/mL of liposomal irinotecan, delivered with gadoteridol using real-time monitoring in the MRI suite. The amended protocol provided a personalized volume at a set concentration based on the volume of each patient’s tumor.

### Procedures

The study procedure was separated into three periods: pre-treatment period, treatment period, and post-treatment/follow-up period. The pre-treatment period included all screening assessments and a 3T brain MRI that occurred at least 21 days prior to the procedure confirming recurrence of HGG. This MRI was also used for trajectory planning using iPlan Flow software (BrainLab AG, Munich, Germany) in consultation with neurosurgery, neuro-oncology, and neuroradiology.

The treatment period, which started 48 h prior to the procedure date, confirmed the established trajectory plan via a second MRI done within 2 days before CED to finalize the trajectory and infusion plan. Next, on the day of infusate delivery, catheter placements and CED infusions were performed in an intraoperative MRI suite (GE 3T MR750W, General Electric Healthcare, Waukesha, WI). A frameless image-guided stereotactic system (VarioGuide, Brainlab AG) was used. Nine fiducial markers were placed on the scalp, and the head was secured with an MR-compatible head clamp (Integra; Princeton, New Jersey). Patients were positioned supine with the head turned. T1- and T2-weighted images were obtained after patient positioning and head immobilization. Preoperative images were used for registration and then fused to baseline MR images containing the preplanned trajectories. The trajectory plan, including burr hole and cannula positioning, were discussed in the MRI suite to ensure maximal tumor coverage.

Following skin incision, the drill guide was inserted into the VarioGuide system and a burr hole was made using the VarioGuide drill kit. After opening the dura and placing a bone anchor, the primed infusion catheter was inserted into the device guide and advanced to the preselected target. A bone anchor was used to hold the catheter in place.

After MRI to ensure placement confirmation, the infusion (containing liposopomal irinotecan and gadoteridol) was started at 1 µl/min using a standard infusion pump (Medfusion 4000, Smiths Medical). Real time MRI imaging was obtained every 1–10 min. If the infusate was refluxing or not proceeding along the desired trajectory, the catheter was adjusted. The procedure was complete when one of the following criteria were met: full tumor coverage, full administration of the personalized infusate volume, or the MRI suite time slot expired.

### Tumor coverage

First, a 3D region-of-interest (ROI) was segmented on the pre-procedure isotropic 3D T1-weighted post-contrast MRI series to obtain the volume (in ml) of the contrast-enhancing portion of the tumor. The pre-procedure MRI was obtained within 21 days prior to the procedure. Then, a 3D ROI was segmented on the intra-procedural 3D T1-weighted MRI series to obtain the volume of the gadoteridol infused via CED, and calculated the volume (in ml) of the CED infusate. We then calculated a percentage of the CED infusate volume relative to the contrast-enhancing tumor volume by dividing the two volumes.

### Dose-limiting toxicity and dose escalation

The Dose Limiting Toxicity (DLT) period was 30 days after the infusion. Patients were clinically examined on Day 1, Day 7, Day 14 and Day 30 post-procedure. DLT was defined as any grade-3 or higher neurological toxicity felt to be attributable to the CED infusion of liposomal-irinotecan with gadoteridol, as well as any systemic grade-3 or higher hematologic or non-hematologic toxicity. If none of the first 3 patients in the cohort experienced a DLT, 3 additional patients were enrolled at the next concentration level. Patient enrollment was staggered every 3 days for acute toxicity monitoring after the initial CED infusion. Staggering was done at each dose level prior to treating the subsequent patient in that cohort. Toxicity was assessed according to the NCI Common Terminology Criteria for Adverse Events Version 5.0 (CTCAE v5.0). Patients between dose-escalating cohorts were reviewed by the institutional Data Safety and Monitoring Committee (DSMC) at the University of California, San Francisco (UCSF) before proceeding with enrollment.

## Results

Between July 2014 and December 2020, 18 patients had CED procedures were performed. There were 8 screen failures, all due to imaging eligibility parameters. The first nine patients were treated on the original protocol, each with one cannula, all in under 6 h (Supplemental Table 3). The original protocol resulted in small infusate volumes with limited tumor coverage (12–58%; Table 1). There were no DLT’s in group 1; there was only 1 DLT of stroke in group 2 (Supplemental Fig. 1), which was considered to be possibly related to the procedure and the study drug, resulting in expanding the cohort to 6 total patients, with no subsequent DLT. After this, the protocol was amended as described above, which allowed for variation in infusate volume based on tumor size. For the amended protocol, all infusions were performed with a single cannula in less than 6 h; three patients were enrolled at the 20 mg/mL dose, then 40 mg/ml, with voluntarily expansion to a total of 6 patients at 40 mg/ml– with no DLTs. Table 4 shows the tumor volume, volume of distribution (V_d_), volume of infusate (V_i_), and tumor coverage percentage. Tumor coverage was improved in the amended protocol (Table 1). Mean progression-free survival after the CED procedure was 17 weeks, and mean overall survival was 67 weeks (Supplemental Table 4) There were no DLTs or significant adverse events (sAEs) in either protocol cohort. Four adverse events were observed, with two of them (grade 3 stroke and grade 2 encephalopathy) being possibly related to the study drug or procedure (Table 2).

**Table 1:** Radiologic response and percent tumor coverage after CED

ID	Tumor type	Tumor volume	Date of CED	V_d_	V_i_	% tumor coverage	Best response	Progression date	Progression location
CED 01	GBM	2.7	10/17/2014	1.5	1.1	24.3	Stable	1/16/2015	Outside infusion area
CED 02	GBM	3	12/5/2014	1.5	1.2	18.2	Progression	1/5/2015	Outside infusion area
CED 03	GBM	2	2/13/2015	3.2	1.0	21.5	Stable	6/5/2015	Outside infusion area
CED 04	AA	3	5/8/2015	2.2	1.0	43.8	Partial response	7/24/2015	Outside infusion area
CED 06	GBM	1.9	5/15/2015	2.2	1.0	31.9	Progression	6/12/2015	Outside infusion area
CED 08	GBM	3	9/11/2015	1.8	1.1	15.9	Stable	1/7/2016	Outside infusion area
CED 09	GBM	2	2/26/2016	2.1	1.0	51.4	Stable	10/31/2016	Inside infusion area
CED 12	GBM	2	1/20/2017	2.1	1.0	58.2	Stable	4/21/2017	Inside infusion area
CED 13	GBM	3	2/10/2017	1.2	1.2	11.6	Stable	5/12/2017	Outside infusion area
CED 16	GBM	3.4	5/4/2018	2.9	3.7	52.6	Interval decrease	11/6/2018	Inside infusion area
CED 17	GS	5.75	9/14/2018	3.2	5.1	33.1	Progression	10/12/2018	Inside infusion area
CED 18	GBM	5.8	9/21/2018	5.9	7.0	49.2	Stable	12/17/2018	Outside infusion area
CED 21	GBM	1.1	3/29/2019	3.4	2.7	91.1	Stable	11/25/2019	Outside infusion area
CED 22	GBM	3.0	1/31/2020	8.8	5.5	41.9	Stable	9/25/2020	Outside infusion area
CED 23	GBM	3.3	2/21/2020	6.2	10.6	95.0	Reduced enhancement	8/20/2020	Outside infusion area
CED 24	GBM	3	6/26/2020	8.9	6	76.0	Stable	3/23/2021	Outside infusion area
CED 25	GBM	5.1	9/4/2020	7.0	6	93.8	Stable	11/30/2020	Inside infusion area
CED 26	GBM	3.5	11/19/2020	11.9	9.3	74.5	Stable	1/13/2021	Inside infusion area

V_d_ - volume of distribution of infusate, V_i_ - volume of infusate

Patients 1–13 (shaded) were treated on the original protocol while patients 16–26 were treated on the amended protocol

**Table 2:** Table of serious adverse events (AEs)

Patient ID	Tumor Volume	Infusion Volume	Infusate concentration	AE - Attribution - Grade
CED 02	3 cm^3^	1.225 mL	20 mg/mL	Seizure– unrelated (x2)– Gr 2
CED 04	3 cm^3^	1.006 mL	20 mg/mL	Sinus Tachycardia– unrelated– Gr 1
CED 08	3 cm^3^	1.125 mL	20 mg/mL	Stroke– possible– Gr 3
CED 21	1.1 cm^3^	2.73 mL	40 mg/mL	Encephalopathy– possible– Gr 2
CED 21	1.1 cm^3^	2.73 mL	40 mg/mL	ANC increase– unrelated– Gr 3

**Fig. 1:**
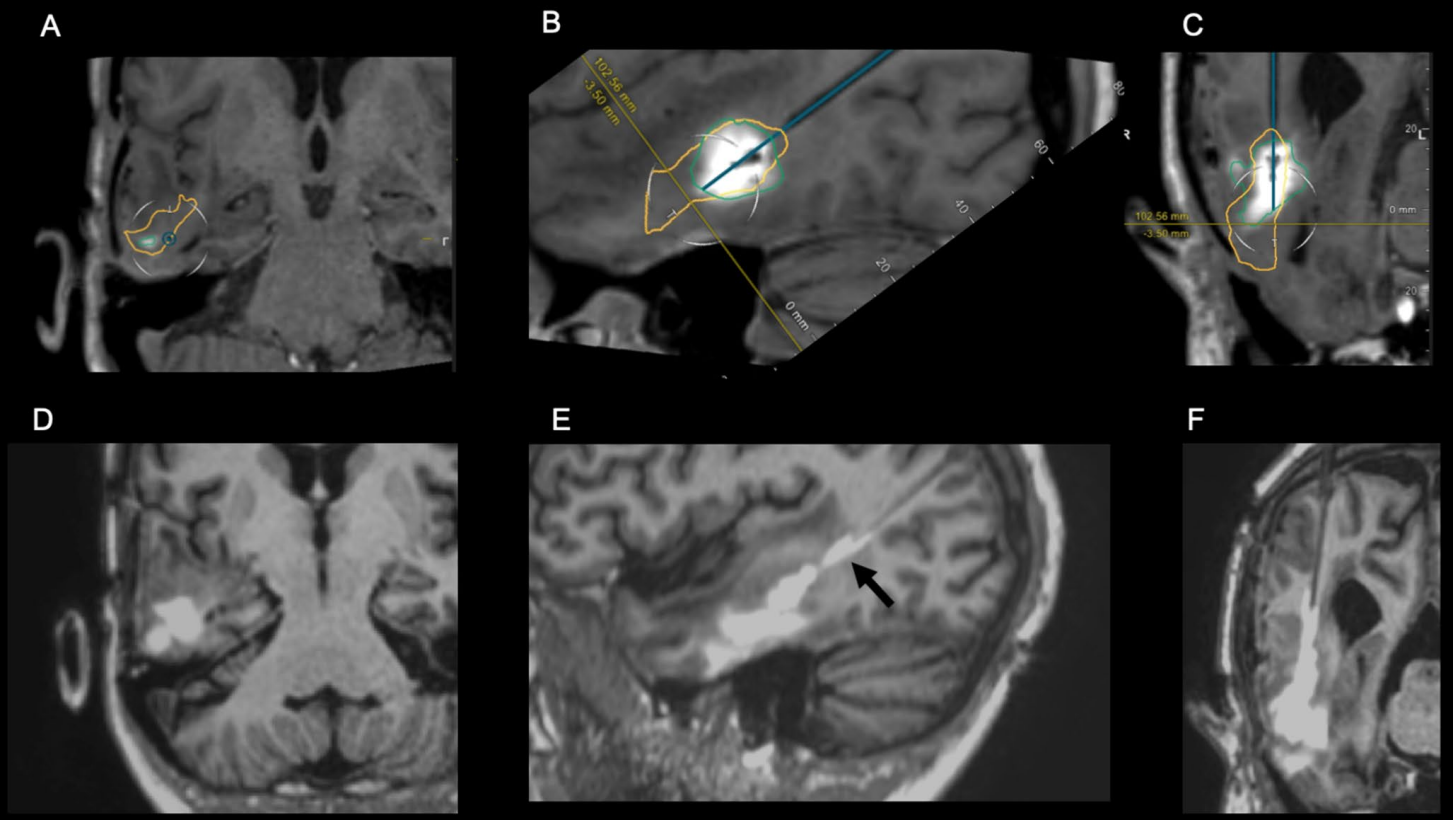
55 year-old patient with right temporal GBM recurrence (CED23). During CED, the area of gadolinium contrast deposition grows to cover 95% of the tumor. Images (**A**-**C**) show initial area of gadolinium contrast deposition on T1-weighted MPRAGE images in coronal oblique (**A**), sagittal oblique (**B**), and coronal oblique (**C**) planes, with green outline indicating the tumor and yellow outline indicating the final infusate coverage. Images (**D**-**F**) show final infusate coverage, with reflux of gadolinium contrast along the catheter tract (black arrow in **E**), that was seen during the procedure and allowed for correction of catheter trajectory

**Fig. 2:**
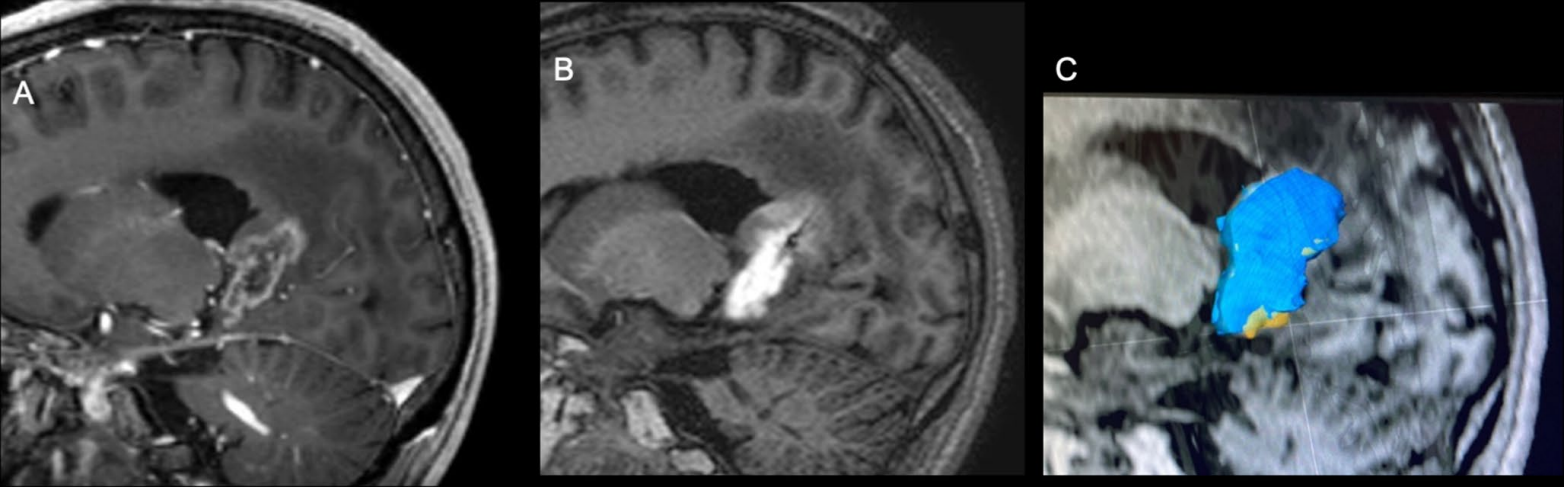
60 year-old patient with left temporal GBM recurrence (CED25) demonstrated by enhancing mass in the left callosal splenium on T1-weighted sagittal MPRAGE image (**A**). During CED, the area of gadolinium contrast deposition grows to cover 94% of the tumor, as demonstrated on intraoperative T1-weighted sagittal MPRAGE image (**B**). Volume segmentation of gadolinium infusate administered during CED (blue) overlaid with volume segmentation of enhancing mass (orange) demonstrates extent of coverage

**Fig. 3:**
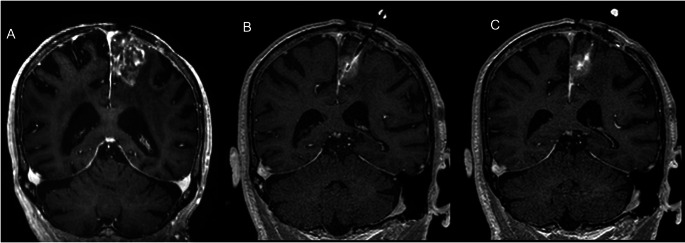
75 year-old man with left superior frontal gyrus GBM recurrence (CED13) demonstrated by enhancing mass on oblique coronal T1-weighted MPRAGE image (**A**). During CED, the infusate volume grows (**B**) until backflow is seen along the cannula and its tract (**C**), with tumor coverage of 12%

Figure 1 shows illustrative images from a CED procedure that achieved 95% tumor coverage and resulted in 6 months of PFS. Reflux of gadolinium contrast along the catheter tract (Fig. 1E) was detected during the procedure and allowed for correction of the catheter trajectory during the procedure. Figure 2 shows illustrative images from a CED procedure that achieved 94% tumor coverage and resulted in 12.4 months of PFS. These cases followed the amended protocol and had large V_i_ with high tumor coverage, in contrast to the procedures performed as part of the original protocol. All cases required catheter administration rate and/or catheter repositioning to maximize coverage Fig. 3.

## Discussion

CED of liposomal irinotecan using real-time imaging with gadolinium contrast in patients with recurrent high-grade glioma was safe in most patients. One patient suffered an ischemic infarct in the ipsilateral cerebral hemisphere > 3 weeks after the CED procedure (Supplemental Fig. 1). This may have been due to drug-induced vasospasm or cannula injury to the lenticulostriate arteries coursing through the treatment volume. However, this adverse event may also have been due to tumor progression causing compression or invasion of the lenticulostriate arteries, as the event occurred > 3 weeks after the procedure. Prior radiation is a risk factor for such vascular events as well.

MRI-guidance allowed for real-time placement of CED cannulas and correction of cannula trajectory to optimize tumor coverage. While the original protocol was limited to small tumors due to inadequate infusate volume, the amended protocol allowed personalized infusate volumes based on tumor size. This treatment modality may address a large gap in available treatment options for recurrent HGG patients [40, 43, 54–56], especially if future studies allow multiple/sequential CED infusions [57] and build on promising CED results [18, 22–48]. The amended protocol provided a personalized volume at a set concentration based on the volume of each patient’s tumor. This design was also employed in other relevant CED trials: (1) CED With Irinotecan Liposome Injection Using Real Time Imaging in Children With DIPG [NCT03086616] (the same study agent was used and up to 10mL of infusate was administered at a concentration of 40 mg/mL); (2) Convection-Enhanced Delivery (CED) of MDNA55 in Adults With Recurrent or Progressive Glioblastoma [NCT02858895], which infused 60 mL of the study agent [58]. Our original study results, along with data from these studies, provided enough evidence to the FDA to allow for expansion of tumor size (1–4 cm diameter), infusion volumes (2–17 ml), infusion rates (up to 50 µl/min), and infusion times (up to 24 h) in an amended protocol as part of this trial.

Several important lessons were learned in conducting this trial. In addition to the volume of infusate, the efficiency of drug delivery via CED depends on optimal catheter trajectory, number of catheters, catheter diameter, infusate flow rate, infusate viscosity, and tissue consistency, which can vary between patients. Potential determinants of treatment response in this trial include the percentage of tumor coverage, V_d_ and V_i_, avoiding “off” target drug deposition, optimizing cannula design [21, 24, 29–32, 59], avoiding backflow [9, 48, 50, 60], and avoiding air bubbles [61]. However, qualitative evaluation of preprocedural, intraprocedural, and postprocedural MRI suggests that higher infusate volumes in the second cohort allowed for better tumor coverage. Infusate flow direction is difficult to predict, as different factors (flow rate, tissue characteristics, resection cavity size, tumor viability, catheter placement) may take precedence at different times during the infusion. Without MRI guidance, infusate often flowed into the resection cavity where interstital pressure was presumably lowest, or surrounding tissue, rather than the tumor [47, 62–64]. In-person, on site decision-making by the team of experts observing infusate flow under real-time MRI guidance was critical for adjustment of catheter trajectories and improved tumor coverage.

While placement of up to four catheters was allowed in the trial protocol, only one catheter was placed because catheter placement required significant time, risk of infection was deemed too high (due to multiple burr holes), or additional MRI time was not available (MRI suites were available for a 12-hour block of time). Single catheter infusions were the most efficient, although the single catheter trajectory was optimized under MRI guidance by advancing or withdrawing the catheter to allow for optimal tumor coverage. This single catheter technique avoided the possibility of catheter trajectories intersecting, and simplified prediction of the infusate path. Thus, the practical execution of CED during this trial improved the precision, reliability, and safety of improved drug delivery into brain tumors [65, 66].

This study has limitations. It remains unclear whether the volume of the infusate, or the total amount of liposomal irinotecan, dictates treatment response. Future studies will need to identify determinants of efficacy and outcomes. Very large tumors could not be treated in one procedural time point as part of this study due to protocol restrictions. Multiple catheters could not be advanced to one treatment location, because of difficulty maneuvering multiple catheters through a single burr hole, so there is room for optimization of the materials and placement of catheters [45, 48, 67, 68] reaching the tumor location. Extensive resource requirements, including material, medical, and scientific expertise was required for this trial, resulting in technical and logistic difficulties. For instance, patient positioning in the MRI scanner did not permit certain preplanned catheter trajectories, which had to be adjusted in real-time using MRI guidance with a highly specialized team at the time of the procedure to optimize tumor coverage. Not all of these resources and experienced professionals may be available in more resource-limited settings.

## Conclusions

Overall, CED using liposomal irinotecan was feasible in 18 patients with recurrent high-grade glioma. One patient had an ischemic infarct that may have been related to the study procedure or drug. While all participants did eventually expire, their PFS and OS were higher than expected for this patient population. Future studies should include multiple infusions over time using different therapeutic agents with larger volumes to ensure optimal tumor coverage, while maintaining the usefulness of the MRI-guided approach for real time decision making during the infusions.

## Electronic supplementary material

Below is the link to the electronic supplementary material.

Supplementary Material 1

Supplementary Material 2

## Data Availability

No datasets were generated or analysed during the current study.
