# Supplementary material for: A phase I study of convection-enhanced delivery (CED) of liposomal-irinotecan using real-time magnetic resonance imaging in patients with recurrent high-grade glioma
Source: J Neurooncol. 2025 Jan 6;172(1):219–27. doi: 10.1007/s11060-024-04904-y (PMC11832582; doi:10.1007/s11060-024-04904-y)
Supplement: Supplementary file 1 — Supplementary Material 1 [file 11060_2024_4904_MOESM1_ESM.docx]

Supplemental Table 1: Original protocol design*. Four cohorts in a 3+3 single dose escalating trial, based on a fixed drug dose and infusion volume.*

| **Group** | **Dose** | **Tumor volume** | **Infusion**  **Volume** | **Irinotecan conc.** |
| --- | --- | --- | --- | --- |
| 1  2  3  4 | 20 mg  40 mg  60 mg  80 mg | 1-4 cm^3^  1-4 cm^3^  2-5 cm^3^  2-6 cm^3^ | 1.0 ml  1.0 ml  1.5 ml  2.0 ml | 20 mg/ml  40 mg/ml  40 mg/ml  40 mg/ml |

Supplemental Table 2: Amended protocol design of a phase 1, 3+3 design, single dose trial with 2 cohorts of ‘personalized’ drug volume but fixed concentration of 20mg/mL in cohort 1 and 40mg/mL of Liposomal irinotecan in Cohort 2, delivered with GAD. *This allowed for tailored volume of Liposomal irinotecan to tumor size at prespecified dose concentrations resulting in variable total dose. Total dose was personalized based on tumor volume, ranging from 20-680 mg of Liposomal irinotecan., with injection*. *Since the total dose of 40 mg had been found to be safe from the original design, subjects were started with concentration cohort 1 at 20 mg/mL with a range of volume of at least 3 mL and a maximum of 17 mL. The patient population and endpoints were unchanged from the original study design. The formula for determining volume of a sphere (e.g., V = 4⁄3πr³) was used as a guide to determine the planned infusate target volume needed to cover the tumor with the continued expectation of Vd/Vi of ~2.*

| **Tumor Diameter (cm)** | **Tumor Volume (cm^3^)** | **Required volume of injection (mL)** | **Dose cohort 1**  **(mg)**  **20 mg/mL** | **Dose cohort 2**  **(mg)**  **40 mg/mlL** |
| --- | --- | --- | --- | --- |
| 1cm | ~0.5cm3 | 2-3 | 40 - 60 | 80 - 120 |
| 2cm | ~4.1cm3 | 3-4 | 60 - 80 | 120-160 |
| 3cm | ~14cm3 | 6-7 | 120-140 | 240-280 |
| 4cm | ~34cm3 | Up to 17 | Up to 340 | Up to 680 |

Supplemental Table 3. Patient demographics.

| **Patient ID** | **Recurrent Diagnosis** | **Age at CED** | **Gender** |
| --- | --- | --- | --- |
| CED 01 | GBM | 53 | M |
| CED 02 | GBM | 70 | M |
| CED 03 | GBM | 57 | M |
| CED 04 | AA | 36 | F |
| CED 06 | GBM | 38 | M |
| CED 08 | GBM | 58 | M |
| CED 09 | GBM | 61 | M |
| CED 12 | GBM | 61 | F |
| CED 13 | GBM | 79 | M |
| CED 16 | GBM | 55 | F |
| CED 17 | GS | 69 | F |
| CED 18 | GBM | 59 | M |
| CED 21 | GBM | 81 | F |
| CED 22 | GBM | 63 | M |
| CED 23 | GBM | 55 | F |
| CED 24 | GBM | 47 | M |
| CED 25 | GBM | 60 | M |
| CED 26 | GBM | 77 | M |

Supplemental Table 4: Progression-free and overall survival after CED. *Patients 1-13 (shaded) were treated on the original protocol while patients 16-26 were treated on the amended protocol*

| **Patient ID** | **Recurrent Diagnosis** | **PFS (weeks)** | **OS (weeks)** |
| --- | --- | --- | --- |
| CED 01 | GBM | 13.00 | 58.43 |
| CED 02 | GBM | 4.43 | 57.71 |
| CED 03 | GBM | 16.00 | 192.86 |
| CED 04 | AA | 11.00 | 77.86 |
| CED 06 | GBM | 4.00 | 19.14 |
| CED 08 | GBM | 16.86 | 39.00 |
| CED 09 | GBM | 35.43 | 145.57 |
| CED 12 | GBM | 13.00 | 50.14 |
| CED 13 | GBM | 13.00 | 323.00 |
| CED 16 | GBM | 26.57 | 248.29 |
| CED 17 | GS | 4.00 | 66.00 |
| CED 18 | GBM | 12.43 | 46.86 |
| CED 21 | GBM | 34.43 | 41.71 |
| CED 22 | GBM | 34.00 | 85.00 |
| CED 23 | GBM | 25.86 | 91.00 |
| CED 24 | GBM | 38.57 | 63.20 |
| CED 25 | GBM | 12.43 | 60.00 |
| CED 26 | GBM | 7.86 | 10.57 |

Supplemental Figure 1. 59 year-old man with right frontal glioblastoma (CED 08) demonstrated by enhancing mass (white arrow) at the deep aspect of the resection cavity on axial T1-weighted MPRAGE image (A). Axial time-of-flight MRA (B) demonstrating relationship of resection cavity to the right internal carotid artery terminus (white arrowhead). Intraprocedural T1-weighted MPRAGE coronal image (C) demonstrating relationship of the CED cannula tip (black arrow) distant from the right carotid terminus (white arrowhead). Axial diffusion-weighted image (D) obtained 25 days after CED procedure demonstrating acute infarct (asterisk) in the right caudate body.
