# Supplementary figures and images for: A phase I study of convection-enhanced delivery (CED) of liposomal-irinotecan using real-time magnetic resonance imaging in patients with recurrent high-grade glioma
Source: J Neurooncol. 2025 Jan 6;172(1):219–27. doi: 10.1007/s11060-024-04904-y (PMC11832582; doi:10.1007/s11060-024-04904-y)

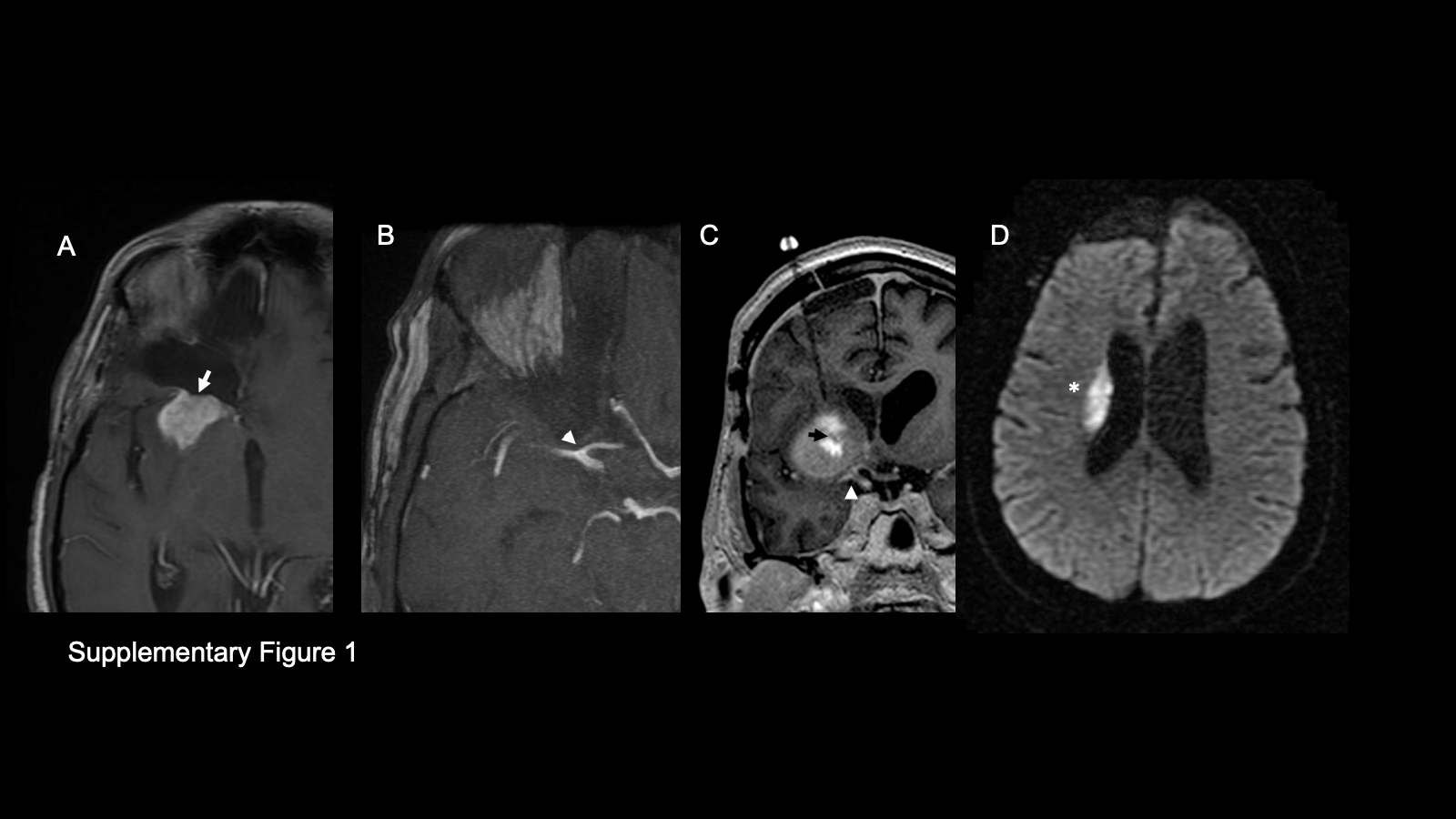

Supplement: Supplementary file 2 — Supplementary Material 2 [file 11060_2024_4904_MOESM2_ESM.tiff]
